# Enhancing Prevention of Injuries in Community youth and adult amateur football teams (EPIC) via implementation support for an exercise-based intervention: study protocol for a type 3 hybrid implementation–effectiveness cluster-randomised controlled trial

**DOI:** 10.1136/bmjopen-2025-102008

**Published:** 2025-08-26

**Authors:** Hanna Lindblom, Sofi Sonesson, Markus Waldén, Ida Åkerlund, Andreas Ivarsson, Martin Hägglund

**Affiliations:** 1 Department of Health, Medicine and Caring Sciences, Unit of Physiotherapy, Linköping University, Linköping, Sweden; 2 Department of Health, Medicine and Caring Sciences, Sport Without Injury ProgrammE (SWIPE), Linköping University, Linköping, Sweden; 3 Capio Ortho Center Skåne, Malmö, Sweden; 4 School of Health and Welfare, Halmstad University, Halmstad, Sweden; 5 Department of Sport Science and Physical Education, University of Agder, Kristiansand, Vest-Agder, Norway

**Keywords:** PREVENTIVE MEDICINE, Implementation Science, SPORTS MEDICINE

## Abstract

**Introduction:**

Injury prevention exercise programmes with multiple components are efficacious in reducing injuries in youth and adult football. However, obtaining high adherence to the programmes over time is an unsolved challenge. Previous studies report that players lack motivation for injury prevention training and that coaches need ongoing support when using these programmes. The overall purpose of this study is to compare the added value of implementation support on the implementation and preventive efficacy of the Knee Control+programme in male and female, youth and adult amateur football teams.

**Methods and analysis:**

This is a type 3 hybrid cluster-randomised controlled trial with adherence to the Knee Control+ injury prevention programme as the primary outcome and preventive efficacy as secondary outcomes. The study will take place during the 2025 football season (April/May to October/November) in Sweden, and we aim to enrol a minimum of 117 teams with players 10 years of age and older. All teams will have access to Knee Control+ via the Swedish Football Association (FA) website and are instructed to use the programme throughout the season. Teams randomised to the intervention group will receive support for implementation of Knee Control+, and the control group will receive no implementation support. This support primarily targets coaches and consists of a smorgasbord of activities and material including physical and digital workshops, leaflets, digital material and site visits to the teams. Codesign with coaches and players was employed during the design of the implementation support. Players report use of Knee Control+ and any injuries or complaints monthly, and coaches report training and use of Knee Control+ weekly, via web-based questionnaires. Adherence to Knee Control+ (primary) and preventive effects on injury incidence and prevalence (secondary) will be compared between groups.

**Ethics and dissemination:**

The study was approved by the Swedish Ethical Review Authority (Dnr 2024-07394-01). Implementation support interventions that are appreciated by the players and/or coaches will be made available and free to use on the Swedish FA website after the study’s conclusion. Fitness trainers will be able to hold workshops and make site visits after the study has been completed.

**Trial registration number:**

Clinicaltrials.gov (NCT06748443).

**STRENGTHS AND LIMITATIONS OF THIS STUDY**

- Broad inclusion of teams of male and female, youth and adult amateur players, as well as their coaches and club representatives, to achieve widespread programme implementation.
- Codesign with coaches and players was employed during the planning of the study and the design of the implementation support interventions, keeping upscaling in mind during the planning stages.
- Comprehensive evaluation of implementation of Knee Control+, behavioural outcomes and injury incidence and prevalence across a whole football season, including a process evaluation.
- Self-reporting by coaches and players of adherence to the training intervention (primary outcome) with no external validation.
- Monthly self-report of injuries (secondary outcome) by players, which potentially excludes some minor injuries from being reported and with no external validation of injury diagnoses.

## Introduction

 Even though multicomponent injury prevention exercise programmes (IPEPs) are proven effective in reducing different types of injuries in youth and amateur football in controlled trials^1^,^7^ and systematic reviews,^8^,^10^ challenges remain in implementing the programmes on a larger scale outside the controlled context of intervention trials. Coaches often modify the programmes’ content or dosage, and few achieve the full prescribed dosage,^11 12^ even though sufficient training dosage is important for maximum effect.^13^,^15^ In the majority of intervention studies targeting injuries in youth team sports, coaches have been presented with the IPEPs at study start during theoretical and/or practical workshops. Afterwards, they have been expected to adopt and use the IPEPs throughout the study, often without receiving any continuous support.^16^ This may be one reason behind implementation challenges.

A committed coach who makes injury prevention a priority is a necessity for the successful implementation of an IPEP.^12^ We have found that coaches and players are generally motivated to use the IPEP Knee Control and have positive beliefs about the programme’s effects.^17 18^ Low player buy-in and players experiencing pain during training, as well as lack of resources (education, time, access to training venues, etc), may be barriers to long-term maintenance.^12 17 19^ Coaches often describe low belief in their ability (self-efficacy) to lead preventive training,^12 18^ and a wish for more support from clubs and football associations (FAs).^12 17^ In addition, few coaches have structured plans for programme use and for working around barriers to maintain programme use when faced with challenges.^18 20^ This suggests that coaches lack action plans (when, where and how plans) and coping plans (compensatory plans for when the original action plan becomes unrealistic or untenable)^21^ and may need further support to achieve continuous programme use.^17^

To address challenges with adherence, the International Federation of Association Football (FIFA) 11 was further developed into FIFA 11+^3^ and exercises were also rescheduled to the end of training.^22^ Similarly, the Knee Control programme^23^ was further developed into Knee Control+ specifically to provide further evidence-based exercise variations and progressions for variability and to fit the youngest players as well as senior players.^24^ Other researchers have used cocreation and worked with important stakeholders when choosing suitable exercises for an IPEP, aiming to achieve optimal adherence. Examples include football,^25^ handball^26^ and Australian football.^27^ The best strategies for supporting programme use from the viewpoint of clubs, coaches and players need to be further studied, and by incorporating behaviour change theories or models. For the present project, we used an already established IPEP, Knee Control+ and employed codesign in the development of an implementation support with the aim of improving programme use.

### Study aim

The overall purpose of this study is to compare the added value of implementation support on the implementation and preventive efficacy of Knee Control+ in male and female, youth and adult amateur football teams. The primary aim is to compare the use of Knee Control+ during a season between teams who receive a preseason digital workshop plus additional implementation support during the season (intervention group (IG)) and teams that receive the preseason digital workshop and access to digital programme material only (control group (CG)). The secondary aim is to compare the preventive efficacy on the rate of lower limb injuries between the two groups. Additionally, we will conduct a process evaluation to assess fidelity and quality of the implementation, and evaluate mechanisms and contextual factors associated with variation in outcomes.

#### Research questions

1. How do use and preventive effect of Knee Control+ differ between teams randomised to an IG receiving implementation support for Knee Control+ from teams randomised to a CG with standard access to digital Knee Control+ programme material?
2. How does coach self-efficacy in using Knee Control+ differ between IG and CG?
3. How do player motivation and player training dosage differ between IG and CG?
4. What are the mediators and moderators that are associated with the use of Knee Control+?
5. How well do the IG and CG adhere to the recommended Knee Control+ usage and how is adherence associated with preventive efficacy?
6. How are the different components of implementation support used by the IG and how are they perceived by clubs, coaches and players, as well as by the fitness trainers who educate coaches in the programme?
7. How do coaches perceive their commitment to lead Knee Control+ training and has this changed after having received implementation support?

## Methods and analysis

### Design

The Enhancing Prevention of Injury in Community football (EPIC) study is a type 3 hybrid cluster-randomised controlled trial^28^ with aspects of implementation being the primary outcome of the study and preventive efficacy being secondary. Football clubs will be randomised in a 1:1 ratio to an IG, which receives additional support for the implementation of Knee Control+, or to a CG without this support. All teams in the same club will be allocated to the same group. Teams in both groups will be recommended to use Knee Control+ throughout the 2025 football season, spanning 7 months from April to October/November. Both groups will have access to the same digital programme material from the Swedish FA and will be introduced to the programme during a digital workshop in the preseason. Block randomisation will be performed at the club level to avoid contamination between teams within the club and will be performed by a statistician. Randomisation will be stratified based on player sex, age and playing level, where applicable. Participants (players and coaches) will not be blinded to group allocation. However, the statistician responsible for the analysis will be blinded to group allocation throughout the analysis. When studying the effects of complex interventions, process evaluation is recommended^29^ and is also planned for this project to assess the support intervention. The protocol aligns with the Standard Protocol Items: Recommendations for Interventional Trials checklist.

### Theoretical framework

**Figure 1:**
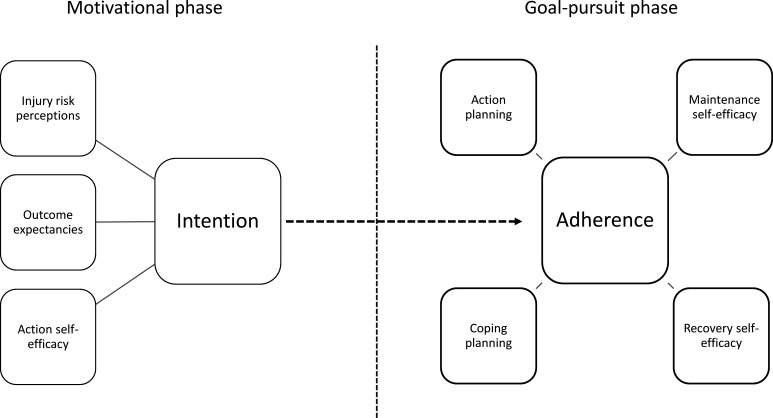
Illustration of the Health Action Process Approach model (adapted from Schwarzer^21^) and how intention for injury prevention training is believed to lead to adherence.

The two-phase behavioural change model, Health Action Process Approach (HAPA)^21^ and the Self-Determination Theory (SDT),^30^ serve as the theoretical base for this study (figures1  2). According to the HAPA model, there is a motivational phase when intention for action is formed and a volitional, goal-pursuit phase when this intention is translated into action. Risk perceptions, outcome expectancies, action self-efficacy and intention are key constructs in the motivational phase, whereas action and coping planning, maintenance and recovery self-efficacy constitute the goal-pursuit phase.^31^ In this study, self-efficacy, action planning and coping planning are specifically targeted in the development of the implementation support and during the evaluation of the study. In previous studies, we have shown that action self-efficacy (belief in one’s ability to use Knee Control+), could be improved among coaches,^18^ as could maintenance and recovery self-efficacy.^17^ The present study has been planned with this in mind. As coaches and players have different roles in regard to injury prevention practices, with the coach usually being the main responsible party and the one who decides whether to use an IPEP, we will only evaluate the intervention effects on HAPA constructs for coaches.

**Figure 2:**
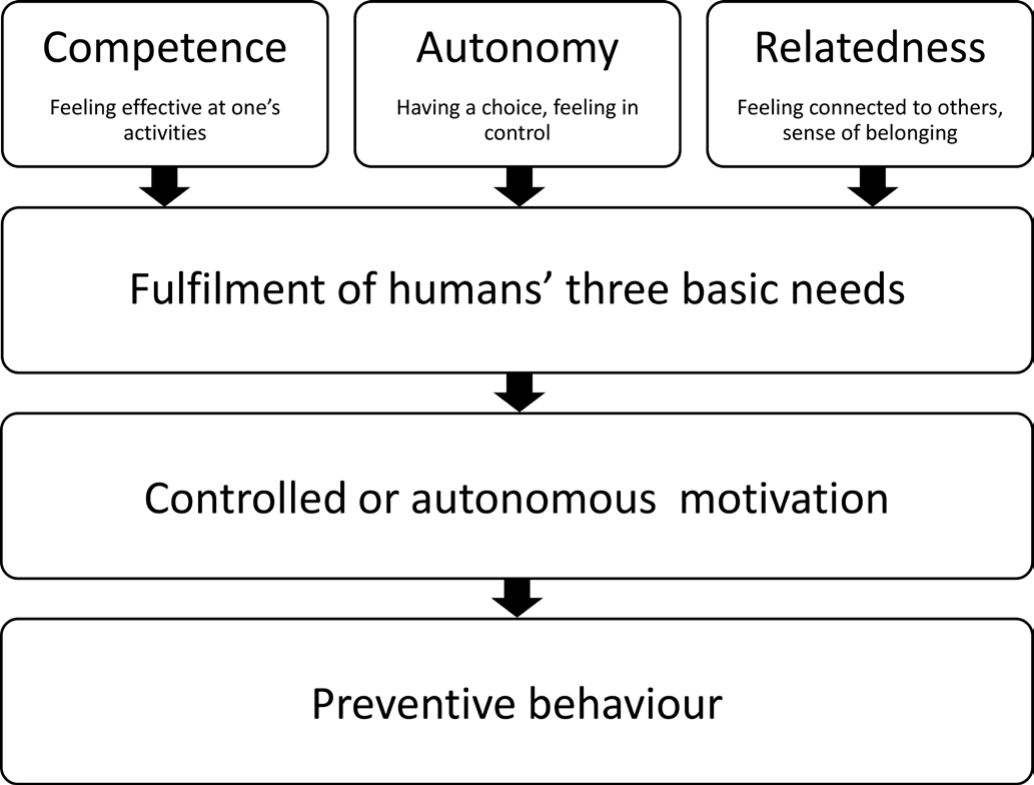
Illustration of the three basic psychological needs of competence, autonomy and relatedness in the Self-Determination Theory^30^ and their influence on motivation in preventive behaviours.

Within the SDT framework, an individual’s motivational regulations for specific behaviours (eg, performing an IPEP) can be classified into three broad categories; that is, autonomous, controlled and amotivation.^30^ When a behaviour is self-initiated and coherent with the individual’s values, it is, according to the theory, regulated by autonomous motives.^30^ Controlled regulation of behaviours is linked to reasons considered external to the individual (eg, perceived pressure from others, external rewards, etc). Individuals who show high levels of autonomous regulation are more likely to demonstrate sustainable and adaptive behaviours.^30^ The fulfilment of the three basic psychological needs—competence, autonomy and relatedness—is suggested to be related to higher levels of autonomous motivation.^32^ In line with the proposed process between basic psychological needs, autonomous motivation and behaviours, research has shown that autonomous motivation mediated the relationship between basic psychological needs and adherence to IPEPs.^33^

### Setting

Male and female, youth and adult amateur football teams, within nine football districts in Sweden (out of 24 total districts), are targeted by the intervention. The nine districts were chosen based on the availability of fitness trainers interested in educating teams coupled with interest from the district FAs in taking part in the study. Knee Control+ (football version) has been available at the Linköping University website since 2021 and was actively spread during the 2021 football season and thereafter spread mostly via diffusion. New films were published by the Swedish FA in December 2024, which were announced via different media.

### Participants

Coaches and players are included in the study. Teams can be included regardless of whether or not they are already using an IPEP at study start. Teams registered to compete in the series for players 10 years or older and with scheduled football training at least twice per week are eligible for inclusion. All eligible players in the team are invited to participate, regardless of whether they have an injury at study start. Players with long-term injuries who do not plan to take part in match play in the 2025 season are not eligible for inclusion.

As part of the process evaluation, club representatives, such as club chairmen, sports directors and youth managers, will also be targeted in the same nine districts, in addition to fitness trainers delivering the interventions to the coaches.

### Recruitment

We contacted representatives for the district FAs during the autumn of 2024 and informed them about the study. Before the 2025 competitive season, we will ask district FAs to advertise study participation on their webpages and social media and to provide the research group with contact information for potentially eligible teams. Club representatives will be informed about the study and asked to forward information to their coaches via email and, if needed, by telephone. Coaches will be contacted predominantly by email, and, if needed, by telephone with information about study participation. Coaches who fulfil the inclusion criteria and who have received information about the study and accept participation will then be asked to supply their players (and for players<15 years, also their legal guardians) with a QR code through which they can access written information about the study and consent to participate before data collection commences. For players<15 years of age, informed consent from their legal guardians will be collected (online supplemental material).

### Interventions

#### Baseline digital workshop—IG and CG

Both groups will be invited to preseason digital workshops (30 min) where the study’s aim and procedures, and information about Knee Control+ will be presented. Knee Control+ digital programme material with films and text describing all exercises will be available at the Swedish FA’s website for all teams to access free of charge. We will describe how to access the programme material and how the programme is intended to be used during the workshop.

#### Additional support—IG only

**Figure 3:**
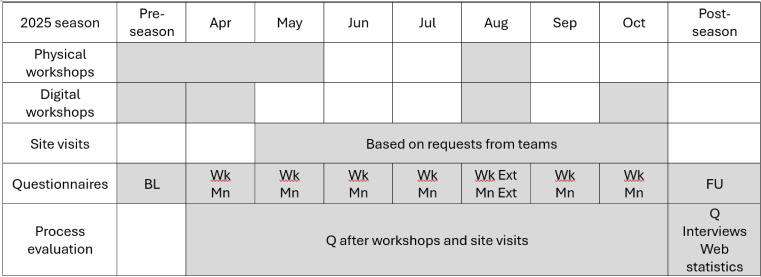
Illustration of the study timeline and significant activities including coaches and players in the intervention group. Enrolment occurs during preseason. Grey cells represent the timing of activities and data collection questionnaires. All activities are optional. BL, baseline; Ext, extended; FU, follow-up; Mn, monthly (for players and coaches); Q, questionnaire; Wk, weekly (for coaches).

For coaches in the IG, the introductory workshop will be extended to also include information about additional support for Knee Control+ programme adoption and use. Coaches will be offered a smorgasbord of implementation support interventions that they can choose from depending on their specific needs: physical workshops, digital workshops, site visits, digital and printed programme material, and posts via social media (table 1 and online supplemental file 2). Fitness trainers will be responsible for physical workshops to train and educate football coaches (described in more detail below). The CG will be offered the same support after the 2025 football season. A timeline of all workshop activities and data collection questionnaires with name coaches and players is presented in figure 3.

**Table 1:** Illustration of the implementation support interventions and the intended effects of each of these interventions for different stakeholders

Intervention component	Intended effect	Outcome
**Targeted at coaches**		
Film about injuries and injury prevention training in football	Increase knowledge of injury prevention	Perceived knowledge assessed through action self-efficacy (HAPA)
Physical workshop at Friskis&Svettis* training venue (or in the club) with several teams in the beginning of the season	Increase coaches’ self-efficacy to lead, progress and maintain KC+ programme use over time Formalisation of action plans	ESES-S Action, maintenance and recovery self-efficacy (HAPA) Existence of action plans
Individualised site visits by fitness trainers from Friskis&Svettis during season	Increase coach self-efficacy to lead, progress and maintain KC+ programme use over time	ESES-S Action, maintenance and recovery self-efficacy (HAPA)
Physical booster workshop at Friskis&Svettis training venue at mid-season	Increase coach self-efficacy to lead, progress and maintain KC+ use Formalisation of action and coping plans	ESES-S Action, maintenance and recovery self-efficacy (HAPA) Existence of action and coping plans
Digital workshops during season (n=3)	Increase coach self-efficacy to lead, progress and maintain KC+ use Formalisation of action and coping plans	ESES-S Action, maintenance and recovery self-efficacy (HAPA) Existence of action and coping plans
Printed leaflet about KC+	Inspiration and reminder to use the programme	KC+ training frequency
Role models (film of coaches leading KC+)	Inspiration to use the programme Support for action planning	KC+ training frequency Existence of action plans
Social media	Reminders to use the programme	KC+ training frequency
**Targeted at players**		
Film about injuries and injury prevention training in football (same as above)	Autonomous motivation to engage in injury prevention training	Single-item scales for basic psychological needs TSRQ
Individualised site visits during season (same as above)	Increase basic needs support and autonomous motivation to engage in injury prevention training	CBS-S Single-item scales for basic psychological needs TSRQ
Printed leaflet about KC+ (same as above)	Autonomous motivation to engage in injury prevention training	Single-items scales for basic psychological needs TSRQ
Positive reinforcement from coaches to players	Improved rating of coaching behaviour and autonomous motivation	CBS-S TSRQ
**Targeted at club staff**		
Printed leaflet about KC+ (same as above)	Inspiration and reminder to use the programme	Whether a leaflet has been published at the club house
Digital preseason workshop	Information and tips for establishing club routines for injury prevention	Presence of written policies at end of season Education in KC+ for coaches

* Friskis&Svettis is a national training organisation of fitness centres in Sweden.

CBS-S, Coaching Behavior Scale for Sport, construct covering technical skills; ESES-S, Swedish version of the Exercise Self-Efficacy Scale; HAPA, Health Action Process Approach; KC+, Knee Control+; TSRQ, Treatment Self-Regulation Questionnaire.

### Education of fitness trainers

Fitness trainers in a nationwide organisation (Friskis&Svettis) will be the primary intervention deliverers and will be trained in the Knee Control+ programme exercises and support interventions to be able to hold workshops at their training venues as well as to make site visits in the teams. Friskis&Svettis has 102 local associations across Sweden, and about 5% of the Swedish population are members of the organisation. Friskis&Svettis offers individual training in the gym as well as different modes of group training. Fitness trainers work pro bono in their spare time, and all have received specific education on how to structure and lead training. The benefits of using fitness trainers as programme educators include: (1) fitness trainers are skilled at delivering training for groups of athletes, (2) fitness trainers across the country can be reached and educated by simple means and at low cost and (3) the use of fitness trainers in a nationwide organisation provides a feasible solution to scale up the support intervention after study completion. The research group, together with heads of the national Friskis&Svettis organisation, developed digital educational material for the fitness trainers and provided the fitness trainers with a structured plan and a PowerPoint presentation to use during coaching workshops. In 2025, fitness trainers will be able to contact the research group by telephone or via email to receive support when needed.

### Data collection

Data will be collected from coaches and players at baseline (pre season) during the 7-month season and at follow-up (post season) through web-based questionnaires distributed via a link sent by email or SMS. All questionnaires will be supplemented by two digital reminders and afterwards we will attempt to contact non-responding players and coaches by phone to complete questionnaires. No imputation of missing data is planned. Primary and secondary outcomes are specified in table 2.

**Table 2:** Outcome measures, measurement points and informants

	Baseline	Weekly	Monthly	Follow-up	Description	Informant
**Primary outcome**						
Use of KC+	x	x		x	Proportion of weeks with: KC+ training at least two times and for players 10–12 years, use of ≥3 of 6 main exercises or for players >12 years, use of ≥4 of 6 main exercises, during these sessions	Coach
**Secondary outcomes**						
**Implementation outcomes**						
Adherence to KC+						
Cumulative utilisation	x	x			Proportion of training sessions where KC+ was used	Coach
Utilisation frequency*	x	x	x	x	N times/week	Coach,player
Duration fidelity	x			x	Minutes spent on KC+ each session	Coach
Utilisation fidelity				x	How KC+ was used at training	Coach,player
				x	Number of and which exercises that were used	Coach,player
**Behavioural outcomes**						
Self-efficacy	x		x	x	HAPA and rated on ESES-S	Coach
Presence of action plans	x		†	x	HAPA question	Coach
Presence of coping plans			†	x	HAPA question	Coach
Autonomous motivation	x			x	TSRQ	Player
Basic psychological needs	x		†	x	Single-items scales for basic psychological needs	Player
Basic need support	x			x	CBS-S	Player
Perception of club support	x			x	GTCS	Coach
**Injury outcomes**						
Incidence of new injuries Monthly prevalence of injuries			x x		OSTRC Questionnaire ≥7 days time-loss injuries (primary injury outcome variable) Time-loss injuries (secondary injury outcome variable) All injuries (secondary injury outcome variable)	Player
Adverse events				x	Three questions on occurrence, type and frequency of any KC+ training-related adverse events	Coach, player

* Utilisation frequency is measured weekly for coaches and monthly for players.

† Questions distributed after the summer break as an extended version of the monthly questionnaire.

CBS-S, Coaching Behavior Scale for Sport, construct covering technical skills; ESES-S, Swedish version of the Exercise Self-Efficacy Scale; GTCS, General Training Climate Scale; HAPA, Health Action Process Approach; KC+, Knee Control+; OSTRC, Oslo Sports Trauma Research Center; TSRQ, Treatment Self-Regulation Questionnaire.

### Baseline questionnaires

For players, at baseline, we will collect data pertaining to demographic information (eg, age, sex, number of years’ experience as a football player), previous and current injuries, current injury prevention training practices, autonomous motivation for injury prevention training, and basic psychological needs.

Autonomous motivation will be evaluated using the Treatment Self-Regulation Questionnaire, which originated from SDT and has been validated also in a sporting context.^34^,^36^ In addition, validated single-item scales assessing satisfaction of three basic psychological needs will be used.^37^ Both questionnaires are rated on 1–7 Likert scale, where 1 is rated for statements in which the respondent finds the statement to be ‘not true at all’ and 7 is reserved for statements that the respondent finds to be ‘very true’. Basic needs support in terms of perceived coaching behaviour relating to the performance of injury prevention training will be evaluated using the Coaching Behavior Scale for Sport and the construct that covers technical skills.^38^ This has been validated in a Swedish context.^39^ Questions from all validated questionnaires will be adapted to an injury prevention context.

For players under 15, parents or legal guardians will be invited to participate as proxies and will respond to the questionnaires together with the child at baseline, monthly and follow-up interviews.

For coaches, we will collect demographic information at baseline about the coaches (age, sex, number of years’ experience as a football coach and as a football player, general coach education and education in injury prevention), previous injuries, their current injury prevention training practices, questions pertaining to motivation and goal pursuit in injury prevention training, including self-efficacy, based on the constructs in the HAPA model and based on the Swedish version of the Exercise Self-Efficacy Scale (ESES-S).^40^,^42^ The questions that build on the HAPA model are rated on 0–10 numerical rating scales, where 0 is the least and 10 the most favourable option. The ESES-S is rated on a 0–10 scale, where 0 is equal to ‘not confident at all’ and 10 is equal to ‘completely confident’. Coaches will also respond to questions about the perceived training climate in their club using the General Training Climate Scale, where questions are rated on a 1–5 Likert scale and 1 is equal to the least and 5 the most favourable option.^43^ The baseline questionnaires will be distributed after the coaches have agreed to participate in the study and before randomisation. One coach per team will respond; preferably the coach who will take the greatest part in Knee Control+ training in each team.

The questions based on the HAPA model have been used in previous studies by the research group^18 20^ and were originally inspired by studies with the IPEP FIFA 11+.^44^ The ESES-S has been validated in a Swedish population of patients with rheumatoid arthritis.^42^ In the present study, questions have been modified to an injury prevention context.

### Questionnaires during the season

Players will receive questionnaires on a monthly basis during the season. In these, players self-report participation in football training and matches (number of athlete exposures) and injury prevention training in the last month, as well as the occurrence of injury in any body location in the past month based on the Oslo Sports Trauma Research Center overuse injury questionnaire .^45^ Continuous data monitoring will be accomplished, and players with inconsistent responses to the questions will be contacted by telephone to clarify their answers. At mid-season, in August, players will receive an extended monthly questionnaire with three single-item scale questions covering basic psychological needs.^37^ Self-reports were chosen in line with international consensus on injury surveillance in sports.^46^

Coaches will receive questionnaires on a weekly basis, where they report on the use of Knee Control+ in terms of training frequency and on how many of the six main exercises they used per session (primary outcome). Each month, the questionnaire will be extended to include questions about action self-efficacy, and at mid-season, two questions about action and coping planning (secondary outcomes).

### Follow-up questionnaires after the season

Similar questions (except demographics and injuries) as at baseline are included in the follow-up questionnaires that will be distributed to players and coaches after the 2025 football season. In addition, coaches and players will respond to questions about the occurrence, type and frequency of any adverse events that they may have experienced in relation to the training intervention Knee Control+. Coaches will also receive questions about self-efficacy, action and coping plans in the follow-up questionnaire.

### Qualitative interviews

In addition to questionnaire-based data, we will conduct qualitative interviews with coaches in the IG with a focus on: (1) their experiences of the implementation support as part of the process evaluation and (2) their self-efficacy, action and coping plans (intervention targets) and whether these have changed over the course of the study. Interviews will be semi-structured using an interview guide, performed over telephone, recorded using a Dictaphone, transcribed verbatim and analysed using inductive qualitative content analysis.

### Process evaluation

A process evaluation may be employed during different stages of the development, evaluation and implementation of complex interventions.^29^ In this project, we plan for a process evaluation based on mixed methods and focusing on whether the implementation support interventions are acceptable, implementable, and scalable and transferable over the long term.^47^ The process evaluation for this project is outlined in table 3 and incorporates quantitative questionnaire data about use of the different implementation support interventions (coaches and players) and attendance at workshops and site visits, as well as how coaches perceived the various components. IG coaches taking part in workshops (physical and/or digital) or site visits, and players attending workshops, will respond to a questionnaire via QR codes immediately after taking part in the intervention with questions about their experiences of the workshop or site visit. At follow-up, we will also ask all participating coaches which parts of the implementation support that they have used or taken part in. In addition, qualitative data from the interviews covering coaches’ experiences in taking part in workshops and from using the other implementation support interventions are included in the process evaluation.

**Table 3:** Illustration of the process evaluation

	Baseline	Weekly	Monthly	Follow-up	Description	Informant
**Process evaluation**						
Participation in physical workshop Participation in site visit Participation in booster workshop Participation in digital workshop 1, 2 or 3 Use of printed material Use of digital material Use of social media Use of KC+ material via the Swedish FA	x*			x x x x x x x x *	N coaches (and n players) who have used different support intervention components	Coach Coach Coach Coach Coach Coach,player Coach Coach,player
Perceptions of the support intervention			†	x	Bespoke questionnaire, NRS 0–10	Coach,player
Perceptions of the support intervention				x	Qualitative interviews	Coach
Web statistics				x	Access to different modules in digital implementation support	Overall use
Club prevention policies and practices				x*	Bespoke questionnaire	Club
Perception about the education of coaches				x	Bespoke questionnaire	Fitness trainers

* The intervention group takes part in all aspects of the process evaluation, the control group takes part in selected aspects.

† Questionnaire to coaches and players after taking part in workshops and site visits.

FA, Football Association; KC+, Knee Control+; NRS, Numeric Rating Scale.

Club representatives will receive a short questionnaire at follow-up including questions about the presence of policies for injury prevention training in the club and whether and how the club has supported injury prevention strategies within the club during the present season.

Experiences from fitness trainers as well as representatives for the fitness organisation (Friskis&Svettis) will be evaluated using a short questionnaire at follow-up focusing on arranging workshops and educating coaches.

To follow-up on the use of digital material at the Linköping University website, we will collect website statistics on the use of the different implementation support interventions.

As mentioned in Moore *et al*,^29^ the external validity of the study may be compromised if stakeholders learn about the results of the process evaluation before study completion. Therefore, workshops and site visits will be evaluated immediately after taking part, whereas the rest of the process evaluation will be accomplished at follow-up after the 2025 football season.

### Statistical methods

#### Sample size calculation

Sample size calculations aimed for the minimum sample required to detect a meaningful difference in the primary outcome between the IG and CG. The unit of observation for the primary outcome is defined as the proportion of weeks with sufficient use of Knee Control+. Sample size calculations were completed using the Shiny cluster-randomised trial (CRT) online tool for binary outcomes, based on a logistic regression model. Calculations assumed three teams per cluster (club); a coefficient of variation equal to 0.9 was applied to account for variation in cluster sizes. An exchangeable correlation structure was assumed, and base case calculations used an intracluster correlation (ICC) of 0.02. Sensitivity analyses considered ICC values between 0.01 and 0.05. Chosen ICC values were guided by empirical studies for binary outcomes.^48^ The estimated proportion of weeks where teams use the Knee Control+ in the CG is 0.5 (50%), and in the IG 0.8 (80%) based on published data.^4 19^ Considering a design effect of 1.04, a cluster size of three teams will allow us to detect a between-group difference in the primary outcome of at least 0.3 (30%) with 80% power at the 5% level of statistical significance, with a total of 29 clusters (85 teams). For the same difference in outcome, varying the ICC results between 0.01 and 0.05 rendered sample sizes between 28 and 30 clusters (83–90 teams). Considering a dropout rate of 30%, we aim to recruit a minimum of 117 teams.

For the main injury outcome, ≥7 days of time-loss injuries, we completed sample size calculations based on a Poisson regression model using the Shiny CRT online tool. Calculations assumed 15 players per team (cluster in the secondary analysis), a coefficient of variation equal to 0.9 was applied to account for variation in cluster sizes. An exchangeable correlation structure was assumed, and base case calculations used an ICC of 0.02. Sensitivity analyses considered ICC values between 0.01 and 0.05. The estimated injury incidence in the CG is 0.2 (20%), and in the IG 0.1 (10%) based on published data.^2 49 50^ Considering a design effect of 1.28, a cluster size of 15 players will allow us to detect a between-group relative difference in the secondary outcome of at least 0.5 (50% injury incidence reduction), with 80% power at the 5% level of statistical significance, with a total of 41 teams (607 players). For the same difference in outcome, varying the ICC results between 0.01 and 0.05 rendered sample sizes between 37 and 54 teams (541–806 players). This equals to a total of 14 clusters (range of 12–18 clusters) at the club level.

#### Analysis

Baseline data are presented descriptively. The primary outcome, the use of Knee Control+, will be analysed at the team level as the proportion of weeks with sufficient use of the programme and compared between IG and CG using logistic regression. The cut-off for sufficient training frequency is set at Knee Control+ training at least twice per week. Since a gradual start of injury prevention training is recommended for younger players by the Swedish FA, for players 10–12 years of age, we deem the use of ≥3 of 6 main exercises to be sufficient, whereas for players>12 years of age, the use of ≥4 of 6 main exercises is deemed sufficient to be defined as ‘use of Knee Control+’. Other measures of implementation will also be compared between groups, predominantly with parametric statistics.

Data on any football-related injury will be collected regardless of the need for care or absence from football training or matches (‘all physical complaints’ being the definition of injury). Injury incidence rate (IR, number of injuries/1000 athlete exposures) and monthly prevalence rate (PR, number of athletes reporting an injury/total number of athletes per month) will be presented with 95% CI, and IR ratios and PR ratios will be calculated and compared between groups (according to intention to treat) using Generalized Linear Models with Poisson distribution, log link, and the natural logarithms of total athlete exposures or total eligible weeks as offset denominator variables.

Qualitative interviews will be analysed using qualitative content analysis and an inductive approach.^51^

### Patient and public involvement

Codesign in the development of the implementation support has been employed, as in similar implementation-effectiveness trials aimed at sports IPEP implementation support.^52^ During the 2024 season, a working group with representatives from coaches and players (for practical reasons, age ≥18) was initiated. This working group (two coaches, one former player) was invited to physical and digital meetings with MH and HL and provided feedback orally and in writing and new ideas during the development of the implementation support and questionnaires for coaches and players. Coaches in the group were invited to test certain aspects of the support intervention with their own players (female youth, female and male youth, respectively) and invite their own players to provide feedback that they forwarded to the research group.

We also pilot tested the workshop concept three times at two Friskis&Svettis venues (two cities) with fitness trainers leading workshops for football coaches during the autumn of 2024 in a district not included in the main study. Participants (coaches and players) received a questionnaire about their experiences of the workshops and potential improvements, and fitness trainers gave verbal evaluation to the research group regarding the outcomes of the workshops. One site visit was also accomplished, and the fitness trainers gave feedback on their experiences to the research group following the site visit. Thereby, we received feedback on the workshop and site visit concepts and from both coaches, players and fitness trainers.

When planning and during the study, we have had and will continue to have regular contact with representatives for football clubs, district FAs, the Swedish FA and Friskis&Svettis to get their strategic input on the work done by the coach and player working groups as well as work specifically with the train the trainers concept and the injury prevention policy development in clubs to ascertain that the smorgasbord of implementation support will be accepted for use by the Swedish FA after the study ends.

Most of the aforementioned stakeholders will mainly be active during the planning of the study, but a few may also be invited to offer their views on the results and the interpretation of results after the 2025 season. After the 2025 season and before upscaling the study, we will also follow up on the workshop strategies with Friskis&Svettis about any changes needed before we open up implementation support for national dissemination.

### Ethics and dissemination

Ethical approval was granted from the Swedish Ethical Review Authority (Dnr 2024-07394-01). Participant consent forms are in Swedish and available from the Swedish Ethical Review Authority on request. All participants will be given a study-specific ID and the list connecting names with IDs will be stored separately. Deidentified data will be used in the analyses. All data will be stored on secure servers at Linköping University, with access available to only a select number of researchers (HL, MH, SS and IÅ). Linköping University and the principal investigator (MH) are owners of the data. Data will be continuously monitored to ascertain high quality, but no specific data monitoring committee is needed. The research group, primarily the principal investigator, will monitor the data and check that the protocol is being followed. Any potential changes to the preregistered study protocol will be reported in the following publications, published on clinicaltrials.gov, and, if necessary, a revised ethical application will be submitted.

This is an interdisciplinary project conducted in a research group with experience from previous studies on the implementation of IPEPs in sport as well as sustainable and healthy participation in sport, also embracing a psychological perspective. This study is one of the few that has incorporated behaviour change theories or models, such as the HAPA and SDT, in the development of coach support interventions for an IPEP.^16^ Even though the Knee Control+ programme material was mainly formed by research, rather than through codesign, we believe that it is important to focus on how the programme is spread and communicated and not only focus on the programme’s content. This study builds on close cooperation with a working group of players and coaches and uses a nationwide sports organisation of fitness trainers to educate coaches and players on the programme, with the aim of facilitating upscaling. Implementation support interventions that are appreciated by the players and/or coaches will be made available and free to use on the Swedish FA website after the study’s conclusion. The digital education for fitness trainers will be accessible also after the study has been finalised and fitness trainers will be able to hold workshops and make site visits after the study. All implementation support was developed based on an intention to be able to use the same interventions, without or with only minor modifications, if/when upscaling after the study ends.

This study protocol will be published in an open-access format. Access to deidentified data may be granted by researchers on reasonable request. Results will be spread by means of scientific publications, conference presentations, and popular science articles and oral presentations. We will also publish a popular science report to distribute among participating clubs and coaches, as well as participating organisations.

## Supplementary material

10.1136/bmjopen-2025-102008 online supplemental file 1

10.1136/bmjopen-2025-102008 online supplemental file 2
