# Supplementary material for: Enhancing Prevention of Injuries in Community youth and adult amateur football teams (EPIC) via implementation support for an exercise-based intervention: study protocol for a type 3 hybrid implementation–effectiveness cluster-randomised controlled trial
Source: BMJ Open. 2025 Aug 26;15(8):e102008. doi: 10.1136/bmjopen-2025-102008 (PMC12382590; doi:10.1136/bmjopen-2025-102008)
Supplement: online supplemental file 1 [file bmjopen-15-8-s001.docx]

**Samtycke vårdnadshavare för spelare yngre än 15 år**

Jag har läst skriftlig information om studien och har haft möjlighet att ställa frågor.

Jag samtycker till att mitt barn deltar i studien *Stöd för användning av skadeförebyggande träningsprogram inom barn-, ungdoms- och seniorfotboll* och vet att deltagande är helt frivilligt.

Jag samtycker till att uppgifter om mitt barn behandlas på det sätt som beskrivs i forskningspersonsinformationen.

| Barnets namn  ___________________________________ | Förening/lag (t ex F11, Linköpings BK)  ___________________________________ |
| --- | --- |
| Barnets födelsenummer (år/månad/dag)  ___________________________________ | __________________________________ |
| Namn vårdnadshavare  ___________________________________ | Namnteckning vårdnadshavare  ___________________________________ |
| Ort och datum  ___________________________________ |  |

| Jag önskar att kontakt för månadsenkät och uppföljningsenkät sker via följande mailadress/mobiltelefonnummer (ange e-post och mobilnummer, antingen till dig som vårdnadshavare eller till ditt barn beroende på vad ni tycker blir bäst) | |
| --- | --- |
| Mail  ____________________________________ | Mobiltelefon  ___________________________________ |
| Mailadressen går till  Vårdnadshavare Barnet | Telefonnumret går till  Vårdnadshavare Barnet |
| Alternativ kontaktväg mail/mobiltelefon  _________________________________________________________________________ | |
