# Supplementary material for: Enhancing Prevention of Injuries in Community youth and adult amateur football teams (EPIC) via implementation support for an exercise-based intervention: study protocol for a type 3 hybrid implementation–effectiveness cluster-randomised controlled trial
Source: BMJ Open. 2025 Aug 26;15(8):e102008. doi: 10.1136/bmjopen-2025-102008 (PMC12382590; doi:10.1136/bmjopen-2025-102008)
Supplement: online supplemental file 2 [file bmjopen-15-8-s002.docx]

# Supplement

## Description of the smorgasbord of different implementation support interventions for the intervention group

### Timeline of implementation support interventions

| 2025 season | Pre-season | April | May | June | July | August | September | October |
| --- | --- | --- | --- | --- | --- | --- | --- | --- |
| Physical workshops | Start-up | | |  |  | Booster |  |  |
| Digital workshops | Action planning | |  |  |  | Coping planning |  | Maintenance |
| Site visits |  |  | Individual site visits based on requests from teams | | | | | |
| Other support | Printed and digital support material, films, leaflets, social media activities | | | | | | | |

### Physical workshops at the beginning of the competitive season

All teams in the intervention group (IG) will be offered participation in physical workshops at the beginning of the competitive season. These workshops will be free of charge and will target both coaches who are new to injury prevention training and those who already use injury prevention training. Coaches will be encouraged to bring 1–2 players, predominantly the team captains. Workshops at the Friskis&Svettis training venues are intended to reach coaches from different clubs at the same time, but a similar set-up can also be arranged in the club’s own venues with coaches from several teams in the same club. Fitness trainers will register coach and player participation in workshops.

The workshops will be two hours in duration, and will be led by a minimum of two fitness trainers and include:

1. short theoretical information about injuries in football and effects of injury prevention exercise programmes, (5 min).
2. a practical training session exemplifying how a training session in the team may be conducted (15–20 min).
3. introduction to the six main exercises in Knee Control+ with a discussion about exercise technique and how the coach can instruct their players in the exercises, determine which level to start at, and how to progress the programme (15 min).
4. practical training with various examples of the six main exercises (45 min).
5. questions and answers and discussion in pairs, where coaches meet and talk with each other, followed by a discussion among the whole group, (30 min).

- how to structure the preventive training to make it feasible for use on a regular basis and over a long period of time,
- common challenges for programme use and ideas for how to work around these,
- support with concrete examples of action planning (how to adopt and use the programme in the team) and for coping planning (how to work around barriers and succeed with maintained use) and ideas for how to cooperate around injury prevention within and between teams,
- coaches will also be supported regarding how to deal with pain during prevention training,

1. Evaluation of the workshop using a QR code at the end of the workshop session (5 min).

### Booster physical workshops at mid-season

After summer break, physical workshops 90 minutes in duration, focusing on progression and variation of exercises and maintained use of Knee Control+ will be offered to the IG teams. Coaches will be encouraged to bring 1–2 players. Before the workshops, coaches will be asked to discuss with their team what they think about the programme and whether the players have any feedback or questions. These booster workshops will be held at Friskis&Svettis’ training venues and will focus more on practical aspects of using the programme. The content will mainly focus on the following:

1. a practical training session exemplifying what a team training session may look like, (15–20 min)
2. practical training with various examples of the six main exercises (30 min)
3. question and answers and discussion in pairs, where coaches will meet and talk with each other, followed by a discussion among the whole group (30 min)
   - how to structure preventive training to make it feasible for use on a regular basis and over a long period of time,
   - common challenges for programme use and ideas for how to work around these (10 min)
   - support with concrete examples for coping planning (how to work around barriers and succeed with maintained use) and ideas for how to cooperate on injury prevention within and between teams,
   - pain during prevention training and how to deal with this will also be discussed,
4. Evaluation of the workshop using a QR code at the end of the workshop session (5 min).

During practical training (points 1 and 2), medium and advanced variations of each main exercise will be used.

### Digital workshops during the season

Three digital workshops (45 minutes each) targeting coaches are planned during the season with varying content. These workshops will be held by members of the research group. Interaction between participating coaches and researchers is encouraged during all workshops. The following content is planned:

1. Workshop 1, pre-season. Focus on supporting the new Knee Control+ user. The workshop includes:
   - suggestions for how to integrate the programme into football training
   - how to choose the level and exercises to begin the programme
   - how to adapt the exercises to individual players with different levels of physical fitness and in the event of an injury or pain
   - how to make training more fun, such as through the use of competitive elements or football-specific exercises

We will emphasise the importance of planning for injury prevention in advance, to share responsibility for injury prevention among team members and offer suggestions on how to engage players and include them in the planning and execution of injury prevention training.

1. Workshop 2, mid-season, after the summer break. The workshop covers:
   - aspects of exercise progression, when and how
   - commonly described barriers for programme use, such as lack of time or of a place to conduct the training and poor weather, and suggestions for how to work around these barriers
   - pain during training
   - motivation from the viewpoint of the Self-Determination Theory and its three main concepts: autonomy, competence, and relatedness and how to support these in players.
   - maintenance of injury prevention after summer break
2. Workshop 3, end of season. The workshop includes:
   - how to maintain motivation for injury prevention; e.g., by progressing and varying training over a longer period of time
   - maintenance during off-season; for example, if players move on to indoor sports (e.g., basketball, handball, floorball) during the winter season
   - possibilities for future contact with the fitness trainers at Friskis&Svettis after the study; e.g., to organise booster workshops or site visits

A QR code will be presented at the end of each digital workshop and all participants will be asked to evaluate the session.

### Site visits

Teams will be supplied with contact information for a Friskis&Svettis venue in their vicinity and the team may request a site visit by a Friskis&Svettis fitness trainer to their team. The coaches will be asked to inform the players about the upcoming site visit a week in advance and preferably also add a post about this on the team’s digital calendar. During this site visit, all coaches and players in the team will take part in the practical training. The site visit includes discussion about tailoring the programme to the team with progression and variation of exercises and individualised feedback to players and coaches based on the specific needs of the team. The duration of the site visits will be approximately one hour together with the whole team, followed by 15 minutes with the coaches. During site visits, one or two fitness trainers will take part (depending on the number of participants). At the end of the visit, a QR code will be presented to the coaches, and they will be asked to evaluate the session. The site visits will be financed by the teams themselves at a low cost.

### Printed and digital support material

A leaflet in digital and printed format with information about the Knee Control+ programme will be distributed to players, parents/legal guardians, coaches, and clubs. This will be distributed to coaches during physical workshops and site visits, to clubs via post, and will be made available on the research webpage for all stakeholders to download. In this leaflet, we emphasise the beneficial effects of using the programme on a regular basis, describe training set-up and dosage, and the intended target groups—players of different ages as well as male and female players.

Another leaflet specifically targets coach leadership and how to try and overcome challenges related to poor player buy-in, focusing on three themes: how to engage the players, how to make Knee Control+ training fun, and leadership. This will be distributed to coaches during physical workshops and available at the research webpage for download.

### Instructional and inspirational films

A film, 15 minutes in duration, about injuries and injury prevention training will be published on the website intending to increase players’ and coaches’ knowledge about injury prevention, the effects of Knee Control+, and how the programme is intended to be used. Coaches will be asked to show the film to the players during the season’s start-up.

For coaches, a short inspirational film will be published with coaches describing injury prevention from their point of view, and giving examples of how to engage players, how to make training fun, as well as of positive leadership. Examples will be shown of teams using injury prevention with different set-ups; as preparation training, integrated into the football training and inclusion of exercises that players experience as fun.

### Social media activities

Teams in the IG will get access to a closed social media account on Instagram (which many teams use to show their activities) that will be used for regular posts during the season for encouragement of injury prevention, suggestions for progression and for integration of preventive training as well as ideas for maintenance of Knee Control+ during off-season. The aforementioned inspirational film for coaches will be posted on this account. Via this account, we will also make targeted posts for example to suggest suitable exercise options during wintertime or poor weather when exercises lying down on the pitch may be less suitable, exercises to use when space or time is limited, or before the summer break. Ideas for how to make players take responsibility for prevention training during these periods will also be given, such as a training bingo game with a prize for the winner, or a challenge to post a photo of yourself after having completed the preventive training. Via this account, we will also spread information about the digital and physical workshops. Interaction and discussion between researchers and coaches and between coaches will be strived for.

### Support for clubs

The clubs are targeted in one of the aforementioned leaflets that can be published on notice boards in the clubhouse. We will also invite club representatives to a digital workshop at pre-season. The leaflets and workshop cover ideas on how to support injury prevention within the club; e.g., how to organise educational workshops, written policies for injury prevention, etc.
