# Supplementary material for: Enhancing Prevention of Injuries in Community youth and adult amateur football teams (EPIC) via implementation support for an exercise-based intervention: study protocol for a type 3 hybrid implementation–effectiveness cluster-randomised controlled trial
Source: BMJ Open. 2025 Aug 26;15(8):e102008. doi: 10.1136/bmjopen-2025-102008 (PMC12382590; doi:10.1136/bmjopen-2025-102008)
Supplement: Supplementary file 3 [file bmjopen-2025-102008.reviewer_comments.pdf]

PEER REVIEW HISTORY

BMJ Open publishes all reviews undertaken for accepted manuscripts. Reviewers are asked to complete a checklist review form and are provided with free text boxes to elaborate on their assessment. These free text comments are reproduced below.

ARTICLE DETAILS

Title (Provisional)

Enhancing Prevention of Injuries in Community (EPIC) youth and adult amateur football players via implementation support for an exercise based intervention—study protocol for a type 3 hybrid implementation–effectiveness cluster randomised controlled trial

Authors

Lindblom, Hanna; Sonesson, Sofi; Waldén, Markus; Åkerlund, Ida; Ivarsson, Andreas; Hagglund, Martin

---

VERSION 1 - REVIEW

**Reviewer**	**1**
**Name**	**Jaitner, Thomas**
**Affiliation**	**TU Dortmund University**
**Date**	**25-Apr-2025**
**COI**	**None**

The study protocol addresses a very important research gap in the field of injury prevention and presents a very comprehensive and ambitious study design to address the problem of low acceptance and adherence to injury prevention programs.

The paper is convincing due to its well-described and well-reasoned methodological approach and also presents the challenges and limitations in a clear and understandable manner. I am very excited to see the results.

One minor comment concerns the evaluation of the workshops, which appears to be presented differently in the text and in table 3. While on page 13 it is stated that the evaluation will take place immediately after the end of the workshop, the information in the table suggests that the evaluation will take place after the end of the study. This should be clarified.

**Reviewer**	**2**
**Name**	**Obërtinca, Rilind**

Affiliation **Saarland University**

Date **29-Jun-2025**

COI **In my review, I have mentioned two of my previously published papers that are highly relevant and important to the topic of this manuscript, which I have cited as references for the authors' consideration.**

Overall Comments

Overall, this is an interesting study protocol on a highly relevant topic for the readership. The authors present their idea concisely and provide a sound rationale for conducting the study. The methods and planned analyses are well described and generally appropriate. However, a few issues need to be addressed and/or clarified further. Firstly, the self-reporting of injuries by players constitutes a significant limitation. Although the authors acknowledge this, they are encouraged to consider strategies to mitigate its impact if feasible at this point of time. Secondly, (mainly) in the introduction, the authors rely heavily on references from their own prestigious research group. While this is surely acceptable considering their wide range of research on this topic, unfortunately in some cases, it leads to selective interpretation in the current version of the manuscript, particularly regarding the efficacy of injury prevention programmes (IPPs). This issue should be addressed for a more balanced presentation.

Specific minor comments

Title

I recommend dividing the title into two shorter sentences. The current version is difficult to follow.

Abstract

First line: "Injury prevention exercise programmes are efficacious in reducing injuries in sports." Firstly, I would suggest to focus in football only. Additionally, this statement is too strong and not supported uniformly by the existing literature, including findings from RCTs and meta-analyses. A more nuanced statement is required here and in the Introduction.

Suggested references:

Meta-analyses:

- Impellizzeri, F. M., McCall, A., & van Smeden, M. (2021). Why methods matter in a meta-analysis: a reappraisal showed inconclusive injury preventive effect of Nordic hamstring exercise. Journal of clinical epidemiology, 140, 111–124. https://doi.org/10.1016/j.jclinepi.2021.09.007

- Obërtinca, R., Hoxha, I., Meha, R., Lama, A., Bimbashi, A., Kuqi, D., Shabani, B., Meyer, T., & der Fünten, K. A. (2023). Efficacy of Multi-Component Exercise-Based Injury Prevention Programs on Injury Risk Among Footballers of All Age Groups: A Systematic Review and

Meta-analysis. Sports medicine (Auckland, N.Z.), 53(4), 837–848. https://doi.org/10.1007/s40279-022-01797-7

RCTs:

- Steffen, K., Myklebust, G., Olsen, O. E., Holme, I., & Bahr, R. (2008). Preventing injuries in female youth football--a cluster-randomized controlled trial. Scandinavian journal of medicine & science in sports, 18(5), 605–614. https://doi.org/10.1111/j.1600-0838.2007.00703.x

- van Beijsterveldt, A. M., van de Port, I. G., Krist, M. R., Schmikli, S. L., Stubbe, J. H., Frederiks, J. E., & Backx, F. J. (2012). Effectiveness of an injury prevention programme for adult male amateur soccer players: a cluster-randomised controlled trial. British journal of sports medicine, 46(16), 1114–1118. https://doi.org/10.1136/bjsports-2012-091277

- Hammes, D., Aus der Fünten, K., Kaiser, S., Frisen, E., Bizzini, M., & Meyer, T. (2015). Injury prevention in male veteran football players - a randomised controlled trial using "FIFA 11+". Journal of sports sciences, 33(9), 873–881. https://doi.org/10.1080/02640414.2014.975736

Methods and Analysis

Line 20: Please specify the start and end months of the study.

Line 21: What is the age range of the players included? Please clarify.

Lines 23–24: Did the teams involved also participate in the original study on the extended version of the Knee Control programme (Lindblom et al., BJSM)?

Line 25: Consider replacing the term “smorgasbord” throughout the manuscript. It may be difficult to interpret for readers unfamiliar with this terminology and is overused.

Line 29: While the authors acknowledge the limitation of monthly self-reported injuries, the rationale for this method needs to be clearly explained below in the methods. Also, how will injury reporting be handled for participants under 18? This must be explicitly stated.

Strengths

Second bullet: Please clarify the role of coaches and players in the co-design process. How was this organized? How was feedback gathered? Were under-18 players involved, and if so, was parental or coach supervision included? These details should be reported in the manuscript.

Limitations

Second bullet: Self-reporting is acknowledged as a limitation, but in this context, it is a major one that could significantly affect the publication potential of the results. Why was real-time reporting not chosen instead? Real-time methods could greatly reduce under-reporting, especially of minor or moderate injuries that may be forgotten after a 30-day period.

Introduction

Lines 5–6: Please focus exclusively on football and remove references to other sports.

The discussion about the efficacy of IPPs should be balanced and more neutrally discussed, acknowledging that the evidence is not univocal (see references above).

Lines 37–40: Consider mentioning the design of the FUNBALL programme here, which is a relevant and football-specific example of co-created injury prevention. Also, included (more) competitive exercises and high cognitive demands in the exercises. More details the authors can find in the following references

- Obërtinca, R., Meyer, T., & Aus der Fünten, K. (2024). Injury prevention in youth football (soccer): a comprehensive description of the development process of the 'FUNBALL' programme. BMJ open sport & exercise medicine, 10(4), e002260. https://doi.org/10.1136/bmjsem-2024-002260

- Obërtinca, R., Meha, R., Hoxha, I., Shabani, B., Meyer, T., & Aus der Fünten, K. (2024). Efficacy of a new injury prevention programme (FUNBALL) in young male football (soccer) players: a cluster-randomised controlled trial. British journal of sports medicine, 58(10), 548–555. https://doi.org/10.1136/bjsports-2023-107388

Study Aim

Line 56: Please remove the phrase “standard practice.” Other studies have also provided continuous support to coaches during the implementation phase.

Methods (General Remark)

The methods are generally well described, since the study has already begun, it is not feasible to incorporate methodological changes at this point.

VERSION 1 - AUTHOR RESPONSE

Reviewer: 1
Dr. Thomas Jaitner, TU Dortmund University
Comments to the Author:
The study protocol addresses a very important research gap in the field of injury prevention and presents a very comprehensive and ambitious study design to address the problem of low acceptance and adherence to injury prevention programs.
The paper is convincing due to its well-described and well-reasoned methodological approach and also presents the challenges and limitations in a clear and understandable manner. I am very excited to see the results.

Author response: Thank you for your kind words.

One minor comment concerns the evaluation of the workshops, which appears to be

presented differently in the text and in table 3. While on page 13 it is stated that the evaluation will take place immediately after the end of the workshop, the information in the table suggests that the evaluation will take place after the end of the study. This should be clarified.

Author response: Good point, we agree that this may be confusing. Coaches and players will respond to questionnaires about their experiences of the workshops and site visits immediately after having taken part. At follow-up after the season, all coaches will receive questions about which parts of the intervention support that they have used or taken part in (as not all coaches will have used all components). We have clarified this in the text by adding the following sentence on **page 14, line 381:**

At follow-up, we will also ask all participating coaches which parts of the implementation support that they have used or taken part in.

Reviewer: 2
Dr. Rilind Obërtinca, Saarland University
Comments to the Author:
Overall Comments
Overall, this is an interesting study protocol on a highly relevant topic for the readership. The authors present their idea concisely and provide a sound rationale for conducting the study. The methods and planned analyses are well described and generally appropriate.

Author response: Thank you for your kind words!

However, a few issues need to be addressed and/or clarified further. Firstly, the self-reporting of injuries by players constitutes a significant limitation. Although the authors acknowledge this, they are encouraged to consider strategies to mitigate its impact if feasible at this point of time.

Author response: We agree that this may be a limitation, but this is in line with international consensus for collecting data in prospective injury surveillance studies (Bahr et al., 2020, International Olympic Committee consensus statement: methods for recording and reporting of epidemiological data on injury and illness in sport 2020 (including STROBE Extension for Sport Injury and Illness Surveillance (STROBE-SIIS)).

Given the current study design, it was not feasible to use medical personnel to collect data. As the study is already ongoing, unfortunately, we cannot change any procedures. Additionally, we cannot change study procedures considering that the Swedish Ethical Review Authority have approved these.

Secondly, (mainly) in the introduction, the authors rely heavily on references from their own prestigious research group. While this is surely acceptable considering their wide

range of research on this topic, unfortunately in some cases, it leads to selective interpretation in the current version of the manuscript, particularly regarding the efficacy of injury prevention programmes (IPPs). This issue should be addressed for a more balanced presentation.

Author response: Good point. We have modified the first sentence in the abstract and introduction and now specifically describe the effects of injury prevention programmes in youth football and multi-component injury prevention programmes, **page 4, line 82**:

Even though multi-component injury prevention exercise programmes (IPEPs) are proven effective in reducing different types of injuries in youth and amateur football in controlled trials,[1-7] and systematic reviews,[8-10] challenges remain in implementing the programmes on a larger scale outside the controlled context of intervention trials.

Specific minor comments

Title

I recommend dividing the title into two shorter sentences. The current version is difficult to follow.

Author response: Thank you for this suggestion. We have updated the title to the following:

Enhancing Prevention of Injuries in Community (EPIC) youth and adult amateur football players via implementation support for an exercise based intervention—study protocol for a type 3 hybrid implementation–effectiveness cluster randomised controlled trial

Abstract

First line: “Injury prevention exercise programmes are efficacious in reducing injuries in sports.” Firstly, I would suggest to focus in football only. Additionally, this statement is too strong and not supported uniformly by the existing literature, including findings from RCTs and meta-analyses. A more nuanced statement is required here and in the Introduction.

Good point. We have modified the first sentence in the abstract and now specifically describe the effects of injury prevention programmes in youth football:

Injury prevention exercise programmes with multiple components are efficacious in reducing injuries in youth and adult football.

Suggested references:

Meta-analyses:

- Impellizzeri, F. M., McCall, A., & van Smeden, M. (2021). Why methods matter in a meta-analysis: a reappraisal showed inconclusive injury preventive effect of Nordic

hamstring exercise. Journal of clinical epidemiology, 140, 111–124. https://doi.org/10.1016/j.jclinepi.2021.09.007
- Obërtinca, R., Hoxha, I., Meha, R., Lama, A., Bimbashi, A., Kuqi, D., Shabani, B., Meyer, T., & der Fünten, K. A. (2023). Efficacy of Multi-Component Exercise-Based Injury Prevention Programs on Injury Risk Among Footballers of All Age Groups: A Systematic Review and Meta-analysis. Sports medicine (Auckland, N.Z.), 53(4), 837–848. https://doi.org/10.1007/s40279-022-01797-7

RCTs:
- Steffen, K., Myklebust, G., Olsen, O. E., Holme, I., & Bahr, R. (2008). Preventing injuries in female youth football--a cluster-randomized controlled trial. Scandinavian journal of medicine & science in sports, 18(5), 605–614. https://doi.org/10.1111/j.1600-0838.2007.00703.x
- van Beijsterveldt, A. M., van de Port, I. G., Krist, M. R., Schmikli, S. L., Stubbe, J. H., Frederiks, J. E., & Backx, F. J. (2012). Effectiveness of an injury prevention programme for adult male amateur soccer players: a cluster-randomised controlled trial. British journal of sports medicine, 46(16), 1114–1118. https://doi.org/10.1136/bjsports-2012-091277
- Hammes, D., Aus der Fünten, K., Kaiser, S., Frisen, E., Bizzini, M., & Meyer, T. (2015). Injury prevention in male veteran football players - a randomised controlled trial using "FIFA 11+". Journal of sports sciences, 33(9), 873–881. https://doi.org/10.1080/02640414.2014.975736

Author response: Thank you for these suggestions. We have added those most relevant to the present context (Steffen et al., 2008, Owoeye et al., 2012, van Beijsterveldt et al., 2012), one of the suggested meta-analyses (Obërtinca et al 2023) and additional ones (Thorborg et al., 2017, Bullock et al., 2025).

Methods and Analysis

Line 20: Please specify the start and end months of the study.

Author response: We have added the start and end months as suggested.

Line 21: What is the age range of the players included? Please clarify.

Author response: The lower age limit was 10 years, which we have clarified as suggested. We had no upper age limit.

Lines 23–24: Did the teams involved also participate in the original study on the extended version of the Knee Control programme (Lindblom et al., BJSM)?

Author response: No, that study was carried out in another football district, in 2020, and only with players 15 years and older so there is no overlap between studies.

Line 25: Consider replacing the term “smorgasbord” throughout the manuscript. It may be difficult to interpret for readers unfamiliar with this terminology and is overused.

Author response: Thank you for the suggestion. We have kept the “smorgasbord” description at first mention but otherwise replaced it with “implementation support interventions” throughout the manuscript.

Line 29: While the authors acknowledge the limitation of monthly self-reported injuries, the rationale for this method needs to be clearly explained below in the methods. Also, how will injury reporting be handled for participants under 18? This must be explicitly stated.

Author response: Good point. We have elaborated a bit more in the manuscript about the reasons for choosing monthly self-reports, **page 13, line 345:**

Self-reports were chosen in line with international consensus on injury surveillance in sports.[48].

Regarding injury reporting for players under 15, we invite their legal guardians to respond to all questionnaires together with their children and they could also decide which e-mail address or phone number that the questionnaires are sent to. This is described on **page 13, line 315:**

For players under 15, parents or legal guardians will be invited to participate as proxies and will respond to the questionnaires together with the child at baseline, monthly, and follow-up interviews.

Strengths

Second bullet: Please clarify the role of coaches and players in the co-design process. How was this organized? How was feedback gathered? Were under-18 players involved, and if so, was parental or coach supervision included? These details should be reported in the manuscript.

Author response: We agree that this is important. The structure of the co-design is described on **page 18, line 471** and we also clarified that coaches and player provided verbal and written feedback regarding the implementation support interventions and the questionnaires:

This working group (two coaches, one former player) was invited to physical and digital meetings with MH and HL and provided feedback orally and in writing and new ideas during the development of the implementation support and questionnaires for coaches and players. Coaches in the group were invited to test certain aspects of the support intervention with their own players (female youth, female and male youth, respectively)

and invite their own players to provide feedback that they forwarded to the research group.

We also pilot tested the workshop concept three times at two Friskis&Svettis venues (two cities) with fitness trainers leading workshops for football coaches during autumn 2024 in a district not included in the main study. Participants (coaches and players) received a questionnaire about their experiences of the workshops and potential improvements and fitness trainers gave verbal evaluation to the research group regarding the outcomes of the workshops. One site visit was also accomplished, and the fitness trainers gave feedback on their experiences to the research group following the site visit. Thereby, we received feedback on the workshop and site visit concepts and from both coaches, players and fitness trainers.

Limitations
Second bullet: Self-reporting is acknowledged as a limitation, but in this context, it is a major one that could significantly affect the publication potential of the results. Why was real-time reporting not chosen instead? Real-time methods could greatly reduce under-reporting, especially of minor or moderate injuries that may be forgotten after a 30-day period.

Author response: We acknowledge that real-time reporting of injuries, and ideally also validation by medical personnel, could increase the validity of injury reporting. However, the current procedure was chosen for its high feasibility and adapted to the study budget and resources and is also in line with international consensus (Bahr et al., 2020). The study methodology has been approved by the Swedish Ethical Review Authority and no changes to the procedures of injury reporting can be made while the study is ongoing.

Introduction
Lines 5–6: Please focus exclusively on football and remove references to other sports. The discussion about the efficacy of IPPs should be balanced and more neutrally discussed, acknowledging that the evidence is not univocal (see references above).

Author response: Thank you for the suggestion. We have carefully looked through the introduction and removed references to other sports. As mentioned previously, we also modified the first sentence about the efficacy of IPPs.

Lines 37–40: Consider mentioning the design of the FUNBALL programme here, which is a relevant and football-specific example of co-created injury prevention. Also, included (more) competitive exercises and high cognitive demands in the exercises. More details the authors can find in the following references
- Obërtinca, R., Meyer, T., & Aus der Fünten, K. (2024). Injury prevention in youth football

(soccer): a comprehensive description of the development process of the 'FUNBALL' programme. BMJ open sport & exercise medicine, 10(4), e002260. https://doi.org/10.1136/bmjsem-2024-002260
- Obërtinca, R., Meha, R., Hoxha, I., Shabani, B., Meyer, T., & Aus der Fünten, K. (2024). Efficacy of a new injury prevention programme (FUNBALL) in young male football (soccer) players: a cluster-randomised controlled trial. British journal of sports medicine, 58(10), 548–555. https://doi.org/10.1136/bjsports-2023-107388

Author response: Good idea, we have added one of the references.

Study Aim
Line 56: Please remove the phrase “standard practice.” Other studies have also provided continuous support to coaches during the implementation phase.

Author response: Good suggestion. We have removed the suggested phrase.

Methods (General Remark)
The methods are generally well described, since the study has already begun, it is not feasible to incorporate methodological changes at this point.

Author response: Thank you and we agree. The study is running and we cannot make any changes to the study conduct.
